# A prospective study of effects of psychological factors and sleep on obstetric interventions, mode of birth, and neonatal outcomes among low-risk British Columbian women

**DOI:** 10.1186/1471-2393-12-78

**Published:** 2012-08-03

**Authors:** Wendy A Hall, Kathrin Stoll, Eileen K Hutton, Helen Brown

**Affiliations:** 1 University of British Columbia School of Nursing, T201, 2211 Westbrook Mall, Vancouver, British Columbia, Canada, V6T 2B5; 2 Research Associate, Division of Midwifery, University of British Columbia Department of Family Practice, B. 54, 2194 Health Sciences Mall, Vancouver, British Columbia, Canada, V6T 1Z3; 3 Midwifery Education Program, McMaster University, Hamilton, ON, Canada

**Keywords:** Childbirth fear, Sleep deprivation, Fatigue, Anxiety, Obstetrical interventions, Neonatal outcomes

## Abstract

**Background:**

Obstetrical interventions, including caesarean sections, are increasing in Canada. Canadian women’s psychological states, fatigue, and sleep have not been examined prospectively for contributions to obstetric interventions and adverse neonatal outcomes.

Context and purpose of the study: The prospective study was conducted in British Columbia (BC), Canada with 650 low-risk pregnant women. Of those women, 624 were included in this study. Women were recruited through providers’ offices, media, posters, and pregnancy fairs. We examined associations between pregnant women’s fatigue, sleep deprivation, and psychological states (anxiety and childbirth fear) and women’s exposure to obstetrical interventions and adverse neonatal outcomes (preterm, admission to NICU, low APGARS, and low birth weight).

**Methods:**

Data from our cross-sectional survey were linked, using women’s personal health numbers, to birth outcomes from the Perinatal Services BC database. After stratifying for parity, we used Pearson’s Chi-square to examine associations between psychological states, fatigue, sleep deprivation and maternal characteristics. We used hierarchical logistic regression modeling to test 9 hypotheses comparing women with high and low childbirth fear and anxiety on likelihood of having epidural anaesthetic, a caesarean section (stratified for parity), assisted vaginal delivery, and adverse neonatal outcomes and women with and without sleep deprivation and high levels of fatigue on likelihood of giving birth by caesarean section, while controlling for maternal, obstetrical (e.g., infant macrosomia), and psychological variables.

**Results:**

Significantly higher proportions of multiparas, reporting difficult and upsetting labours and births, expectations of childbirth interventions, and health stressors, reported high levels of childbirth fear. Women who reported antenatal relationship, housing, financial, and health stressors and multiparas reporting low family incomes were significantly more likely to report high anxiety levels. The hypothesis that high childbirth fear significantly increased the risk of using epidural anaesthesia was supported.

**Conclusions:**

Controlling for some psychological states and sleep quality while examining other contributors to outcomes decreases the likelihood of linking childbirth fear anxiety, sleep deprivation, and fatigue to increased odds of caesarean section. Ameliorating women’s childbirth fear to reduce their exposure to epidural anaesthesia can occur through developing effective interventions. These include helping multiparous women process previous experiences of difficult and upsetting labour and birth.

## Background

Birthing women in British Columbia (BC) have one of the highest rates of obstetrical intervention in Canada. Of 44,508 women giving birth in 2010 in BC [1], 22% had primary caesarean sections; among women having vaginal birth, 30% had epidural anaesthesia [2], an intervention that has been associated with increased risk for negative effects on breastfeeding initiation [3,4] and duration [5]. With a caesarean section rate at almost double the 15% rate recommended by the World Health Organization [6], health care professionals are alarmed at the rates of caesarean sections and other childbirth interventions in British Columbia [7,8]. Caesarean sections pose increased physical risks for mothers and infants, including extended hospital stays and readmissions [9-12].

Investigations have focused on contributions of health care practices and health care providers to obstetrical interventions [7,8,13]. Empirical data from other countries has supported contributions of women’s psychological status to obstetric interventions [14-22]. Canadian studies have examined contributions of health care practices to labour and birth interventions; however, none have explored independent effects of specific psychological states, e.g. childbirth fear, on interventions and outcomes, while controlling for other psychological states, e.g. anxiety.

### Previous Work

This study builds on previous work, where we explored measures of childbirth fear, sleep deprivation, fatigue, and anxiety and their relationships in a sample of pregnant British Columbian women [23]. In a cross-sectional study, we obtained data from 650 women. Using posters in providers’ offices and gathering places, media, and pregnancy and baby fairs, we recruited women from communities across British Columbia (BC) with 150 births or more annually. Eligibility criteria included: pregnant women who resided in BC, could read and speak English, and were between 35 and 39 weeks gestation, with no medical complications during pregnancy (e.g. bleeding, pregnancy-induced hypertension, gestational diabetes). Of those women, 25% reported high levels of childbirth fear and 22% reported sleep deprivation (< 6 hours of sleep per night). Childbirth fear, fatigue, sleep deprivation and anxiety were highly correlated. For example, women with high childbirth fear were more likely to report higher levels of anxiety and fatigue, less sleep hours per night, more daily stressors, and less available help. Higher family income, a first time pregnancy, completed university education, and higher levels of anxiety and fatigue were associated with higher childbirth fear scores, explaining 29% of the variance. The relationships between childbirth fear, fatigue, sleep deprivation, and anxiety raised questions about their potential contributions to obstetrical and neonatal outcomes.

### Psychological States in Pregnancy: Prenatal Anxiety and Childbirth Fear

In a review of 60 studies examining prenatal maternal stress as a composite measure (including state anxiety), Beydoun and Saftlas concluded maternal prenatal stress and infants’ low birth weights (<2500 grams) were positively correlated [24]. For American women, prenatal anxiety has had a strong association with shortened length of gestation, [25], epidural analgesia, unplanned caesarean section [26]; and preterm birth [27] For Swedish mothers, depression/anxiety was positively associated with risk for planned caesarean section and increased length of labour [28]. Fear of childbirth has been associated with requests for elective caesarean deliveries [14,16,18-20,22] and emergency caesarean section, after controlling for obstetrical complications and history of previous caesarean sections [21] but nulliparous and multiparous women have reported differences in childbirth fear [18,29]. For 443 British women, neither fear of childbirth nor anxiety was associated with mode of birth, including emergency or elective caesarean births [30]. Studies have examined childbirth fear and, and in some instances anxiety, without taking fatigue and sleep deprivation into account.

### Prenatal Sleep Deprivation & Fatigue

Given the many sleep disruptions (e.g., fetal movement, heartburn, voiding at night) that occur in late pregnancy, it is not surprising some pregnant women experience high fatigue levels [15,31] or reductions in sleep time [31,32]. In Taiwanese, Swedish, and American samples, fatigue and sleep deprivation have been associated with increased risk of caesarean sections [15,17,33]. American women reporting less total sleep time experienced higher levels of labour pain and more fatigue during labour [34]. Chang and colleagues concluded sleep deprivation is linked to higher levels of pro-inflammatory serum cytokines, which in turn are associated with a higher prevalence of preterm delivery and postpartum depression [35]. Despite these contributions about effects of sleep deprivation on obstetrical outcomes, previous studies have not examined whether fatigue and sleep deprivation contribute to requests for analgesia and obstetrical interventions beyond caesarean sections.

The purpose of the study is to examine prospectively associations between British Columbian women’s fatigue, sleep deprivation, psychological states (anxiety, childbirth fear), and characteristics (e.g. age and stressors) and their exposure to obstetrical interventions (induction, augmentation, epidurals, and any anaesthetic, caesarean section, assisted vaginal delivery), and adverse neonatal outcomes (preterm, admission to NICU, low APGARS, and low birth weight).

## Method

This study uses the data from our prior work [23] and links it to maternal and newborn birth outcomes. We sought access to maternal and newborn data from Perinatal Services BC (PSBC), which maintains a provincial database of all maternal obstetric and newborn outcomes. Using the personal health numbers of the 97% of women enrolled in our prior study who had consented to have their maternal and newborn records linked to their survey data, we were able to examine relationships between women’s childbirth fear, anxiety, sleep deprivation, fatigue and obstetric interventions, mode of birth, and newborn outcomes. Our hypotheses to be tested by logistic regression analysis were developed a priori, based on the literature. Because childbirth fear differs by women’s parity [29], we divided the sample by parity equal 0 and ≥1 to test hypothesis 3. The whole sample was used to test the remaining hypotheses which follow:

1) women with high levels of childbirth fear are more likely to have epidural anaesthetic (controlling for maternal age, parity, infant macrosomia, previous caesarean deliveries, fatigue, anxiety, sleep deprivation, and available support) than those with low/moderate childbirth fear;

2) women with high levels of anxiety are more likely to have epidural anaesthetic (controlling for maternal age, parity, infant macrosomia, previous caesarean deliveries, fatigue, fear of birth, sleep deprivation, and available support) compared to those with low/moderate childbirth anxiety;

3) a) nulliparous women with high childbirth fear are more likely to give birth by caesarean section (controlling for maternal age, infant macrosomia, intent to request caesarean section, fatigue, anxiety, sleep deprivation, and available support) than women with low/moderate childbirth fear; b) multiparous women with high childbirth fear are more likely to give birth by caesarean section (controlling for maternal age, infant macrosomia, previous caesarean deliveries, fatigue, anxiety, history of difficult or upsetting labours and births, sleep deprivation, and available support) than women with low/moderate childbirth fear;

4) women with sleep deprivation (< 6 hours per night) are more likely to give birth by caesarean section (controlling for maternal age, infant macrosomia, previous caesarean deliveries, intent to request caesarean section, anxiety, childbirth fear, and available support) compared to women without sleep deprivation;

5) women with high levels of fatigue are more likely to give birth by caesarean section (controlling for maternal age, infant macrosomia intent to request caesarean section, previous caesarean sections, childbirth fear, anxiety, and available support) than women with low fatigue levels;

6) women with high levels of childbirth fear will be more likely to have an assisted vaginal delivery (controlling for maternal age, infant macrosomia, fatigue, anxiety, sleep deprivation, and available support) compared to women with moderate/low levels of fear;

7) women with high levels of anxiety will be more likely to have an assisted vaginal delivery (controlling for maternal age, infant macrosomia, fatigue, fear of birth, sleep deprivation, and available support) than women with moderate/low levels of anxiety;

8) women with high fear of birth will be more likely to experience adverse neonatal outcomes (controlling for maternal age, fatigue, anxiety, sleep deprivation, and available support) compared to women with moderate/low levels of fear; and

9) women with high levels of anxiety will be more likely to experience adverse neonatal outcomes (controlling for maternal age, fatigue, fear of birth, sleep deprivation, and available support) than women with moderate/low levels of anxiety.

### Procedures

The study was reviewed and approved by the University of British Columbia Behavioural Ethics Review Board (H05-81091) and BC Women’s and Children’s Hospital Ethics Committee (W06-0211). Signed consent forms accompanied completed questionnaires. The research is in compliance with the Helsinki Declaration. In the previous study, we enrolled 650 pregnant women between 35–39 weeks gestation from May 2005 to July 2007 (See 23 for details). For the analysis of obstetrical outcomes, we excluded women who produced twins (n =10 sets), whose data could not be matched to birth records by personal health number (n = 5), and who did not consent to have their birth records linked to the survey data (n = 11) for a total of 624 participants. Women who did not consent to have their birth records linked to survey data did not demonstrate significant differences on any demographic characteristics than women who consented.

### Measures

We collected data about maternal characteristics including: maternal age, relationship status, self-identified ethnicity, education level, family income, number and type of daily stressors, previous adverse labour and birth experiences, type of primary care provider, attendance at childbirth education classes, and intentions regarding cesarean birth or other interventions. Respondents also completed measures for childbirth fear, anxiety, fatigue, and sleep quality. From the PSBC birth records data, we obtained data on the following variables: women’s parity, epidural anaesthesia, any type of anaesthesia, instrumental delivery (forceps and vacuum extraction), any type of caesarean birth (emergent, scheduled), preterm births of less than 37 weeks, infant macrosomia, infant gestation at birth, APGAR scores at 1 and 5 minutes post birth, infant prematurity (< 37 weeks), infant low birth weight, and admissions to levels 2 and 3 neonatal intensive care nurseries. Level 2 and 3 nurseries provide ventilator support for high risk infants.

For the survey, we used the 33 item *Wijma Delivery Expectancy/Experience Questionnaire-A (W-DEQ)* to measure women’s fear of childbirth [36]. Pregnant women rated their expectations about experiences during labour and birth on scale from 1–5. Scores range from 0–165; higher scores indicate increased levels of childbirth fear. We categorized women as belonging in the high fear group if they scored 66 or higher on the W-DEQ, as reported by Zar et al. [37]. The W-DEQ has demonstrated reliability for nulliparous and multiparous women [36].

To measure anxiety, we used *Spielberger’s State Anxiety Inventory (SAI)*, a 20 item measure of current feelings of anxiety [38]. Scores range from 20–80. This tool has demonstrated validity and reliability with the childbearing population [30,39]. Similarly to other investigators [40], we labeled women as having high anxiety if they scored above 40 on the scale.

*Mindell’s sleep questionnaire* assessed the pregnant women’s sleep patterns and disruptions; it provides similar data to sleep diaries [31]. Women with less than 6 hours of sleep have been categorized as sleep deprived [17]; we also characterized women as sleep deprived if they slept less than 6 hours per night (on average, over a two week period).

*The Multidimensional Assessment of Fatigue Scale* (MAF) is a 16 item instrument that assesses fatigue [41]. We used the Global Fatigue Index which omits the change in fatigue item. Higher scores indicate more fatigue. The MAF has demonstrated reliability and construct validity with pregnant women [42].

### Data Analysis

Using Pearson’s Chi-square test, we examined differences in proportions of women by characteristics reported antenatally for high childbirth fear and anxiety levels compared with low/moderate levels and women with and without sleep deprivation, separately for nulliparas and multiparas.

We performed hierarchical logistic regression modeling to test the 9 hypotheses outlined previously. Control variables were entered in step 1 of the model, followed by the psychosocial predictor of interest (step 2). We used the Nagelkerke statistic and associated p value to determine whether the addition of the predictor variables increased the explanatory power of the models [43]. We calculated 95% confidence intervals around the resulting odds ratios (OR). The reference groups were women with low anxiety and low/moderate fear and more than 6 hours of sleep per night. For the dichotomous variable to assess newborn adverse outcome, neonates received a value of 1 if they had any one or all of the following: admission to an NICU level 2 or 3, 5 minute APGAR score < 7, birth weight < 2500 g or gestational age < 37 weeks.

Assuming medium effect sizes and alpha = .05, a sample size of 618 provided excellent power (in excess of .95) for all analyses. For the regression analyses examining women’s outcomes, we used Bonferroni’s correction (divided 0.05 by 7 hypotheses) to accept p < 0.007. We used p < 0.01 as an indicator of significant results for the Chi Square analyses to avoid making a type 1 error due to multiple comparisons. For scales with less than 10% missing data, values were imputed by replacing each value with the mean of observed values for the variable.

## Results

We examined associations between levels of childbirth fear and anxiety, fatigue, sleep deprivation, and maternal characteristics. For our sample of pregnant women, Cronbach’s alphas for the standardized measures ranged from 0.92 – 0.93 [23]. We found women in the high childbirth fear group did not differ significantly from women in the low/moderate fear group in terms of age, income, most stressors, educational attainment, lone parent status, ethnicity, and attendance at childbirth classes (all *p* > .01, See Table 1). More multiparas in the high fear group reported experiencing health as a stressor (*χ*² = 16.564, *df* =12, p < 0.001) and using obstetricians as care providers compared to multiparas in the reference group (*χ*² = 19.45, *df* = 2, p < 0.001).

**Table 1:** Comparison of women with low and high childbirth fear on characteristics by parity (N = 624)

	**NULLIPARAS**			**MULTIPARAS**		
	**Low/moderate fear (N = 278)**	**High fear (N = 93)**		**Low/moderate fear (N = 187)**	**High fear (N) = 65)**	
	% (N)	% (N)	*p*	% (N)	% (N)	*p*
**Maternal characteristics**						
Age > 35	13.3 (37)	16.1 (15)	0.498	31.6 (59)	29.2 (19)	0.727
Family Income						
< $ BC Average	26.6 (74)	21.5 (20)	0.326	37.4 (70)	32.3 (21)	0.459
Education						
No university	44.2 (123)	46.2 (43)	0.738	48.7 (91)	52.3 (34)	0.613
University degree	55.8 (155)	53.8 (50)		51.3 (96)	47.7 (31)	
Lone parent	2.2 (6)	1.1 (1)	0.506	2.1 (4)	0 (0)	0.235
Ethnicity						
Aboriginal	1.5 (4)	2.2 (2)	0.065	1.7 (3)	5.0 (3)	0.363
Caucasian	91.6 (241)	83.0 (73)		90.3 (159)	88.3 (53)	
Asian	6.9 (18)	14.8 (13)		8.0 (14)	6.7 (4)	
Maternity care provider						
Midwife	24.8 (69)	20.4 (19)	0.069	32.1 (60)	10.8 (7)	< 0.001
Family physician	44.2 (123)	35.5 (33)		38.0 (71)	30.7 (20)	
Obstetrician	31.0 (86)	44.1 (41)		29.9 (56)	58.5 (38)	
Attended childbirth classes	83.1 (231)	84.9 (79)	0.676	12.8 (24)	12.3 (8)	0.999
History of difficult labour and birth	NA	NA	NA	46.5 (86)	70.3 (45)	0.001
History of upsetting labour and birth	NA	NA	NA	28.6 (53)	62.5 (40)	< 0.001
**Maternal characteristics**						
Source of stressor - relationships	23.0 (64)	32.3 (30)	0.076	47.3 (88)	60.9 (39)	0.060
Source of stressor – finances	48.6 (135)	51.6 (48)	0.610	47.8 (89)	62.5 (40)	0.043
Source of stressor – housing	18.0 (59)	24.7 (23)	0.157	14.5 (27)	28.1 (18)	0.015
Source of stressor – employment	26.3 (73)	38.7 (36)	0.023	20.4 (38)	32.8 (21)	0.044
Source of stressor – education	7.9 (22)	2.2 (2)	0.050	4.8 (9)	9.4 (6)	0.187
Source of stressor – health	22.3 (62)	35.5 (33)	0.012	18.3 (34)	43.8 (28)	< 0.001
Requesting a caesarean section	1.1 (3)	3.2 (3)	0.155	11.3 (21)	16.9 (11)	0.241
Requesting a caesarean section, excluding women with a previous caesarean section	1.1 (3)	3.2 (3)	0.155	1.4 (2)	2.5 (1)	0.641
Expecting obstetric interventions, excluding women with a previous caesarean section	14.1 (39)	18.5 (17)	0.308	7.2 (10)	30.0 (12)	< 0.001

A significantly higher proportion of multiparas with a history of difficult and upsetting labour and birth reported high levels of childbirth fear (*χ*² = 10.827, *df* = 1, *p* = 0.001; *χ*² = 23.286, *df* = 1, *p* < 0.001). Higher proportions of multiparas expecting to have obstetric interventions during labour and birth (*χ*² = 16.767, *df* = 1. *p* < 0.001) also reported high levels of childbirth fear. Women with high childbirth fear did not request a caesarean section more often than women with low/moderate childbirth fear (see Table 1).

Significantly fewer multiparas over the age of 35 (χ² = 14.31, *df* = 1, *p* < 0.001) reported high levels of anxiety compared with multiparas who were younger. Significantly more multiparas with a family income below the provincial average ($ 60,000; χ² = 6.44, *df* = 1, *p* = 0.01) reported high anxiety levels compared with those who had incomes above the provincial average. Compared to women reporting low anxiety, larger proportions of nulliparas and multiparas with high anxiety reported stressors (See Table 2). Sleep deprivation was associated with health-related stressors among nulliparas, but not multiparas (*χ*² = 22.054, *df* = 1, *p* < 0.001).

**Table 2:** Comparison of women with low and high anxiety on characteristics by parity (N = 624)

	**NULLIPARAS**			**MULTIPARAS**		
	**Low anxiety**	**High anxiety**		**Low anxiety**	**High anxiety**	
	**(N = 279)**	**(N = 93)**		**(N = 164)**	**(N = 88)**	
	% (N)	% (N)	*p*	% (N)	% (N)	*p*
**Maternal characteristics**						
Age > 35	15.1 (42)	11.8 (11)	0.441	39.0 (64)	15.9 (14)	< 0.001
Family Income						
< $ BC Average	24.4 (68)	28.0 (26)	0.491	30.5 (50)	46.6 (41)	0.010
Education						
No university	42.7 (119)	50.5 (47)	0.185	44.5 (73)	59.1 (52)	0.027
University degree	57.3 (160)	49.5 (46)		55.5 (91)	40.9 (36)	
Lone parent	1.8 (5)	2.2 (2)	0.826	1.8 (3)	1.1 (1)	0.675
Ethnicity						
Aboriginal	1.1 (3)	3.4 (3)	0.343	1.3 (2)	5.1 (4)	0.218
Caucasian	90.2 (239)	87.4 (76)		91.1 (143)	87.3 (69)	
Asian	8.7 (23)	9.2 (8)		7.6 (12)	7.6 (6)	
Maternity care provider						
Midwife	26.5 (74)	15.1 (14)	0.026	29.9 (49)	20.5 (18)	0.107
GP	42.3 (118)	40.9 (38)		37.2 (61)	34.1 (30)	
Obstetrician	31.2 (87)	44.1 (41)		32.9 (54)	45.5 (40)	
Attended childbirth classes	83.5 (233)	83.9 (78)	0.936	12.2 (20)	13.6 (12)	0.743
History of difficult labour	NA	NA	NA	51.2 (83)	55.2 (48)	0.553
History of upsetting labour	NA	NA	NA	34.6 (56)	42.5 (37)	0.216
**Maternal characteristics**						
Source of stressor - relationships	21.1 (59)	37.6 (35)	0.002	42.3 (69)	66.7 (58)	< 0.001
Source of stressor – finances	45.2 (126)	61.3 (57)	0.007	43.6 (71)	66.7 (58)	< 0.001
Source of stressor – housing	15.1 (42)	33.3 (31)	< 0.001	12.9 (21)	27.6 (24)	0.004
Source of stressor – employment	26.2 (73)	38.7 (36)	0.021	17.8 (29)	34.5 (30)	0.003
Source of stressor – education	5.4 (15)	9.7 (9)	0.144	3.7 (6)	10.3 (9)	0.035
Source of stressor –health	20.8 (58)	39.8 (37)	< 0.001	17.2 (28)	39.1 (34)	< 0.001
Requesting a caesarean section	1.1 (3)	3.2 (3)	0.154	9.2 (15)	19.3 (17)	0.022
Requesting a caesarean section, excluding women with a previous caesarean section	1.1 (3)	3.2 (3)	0.154	1.6 (2)	1.8 (1)	0.950
Expecting obstetric interventions, excluding women with a previous caesarean section	13.7 (38)	19.6 (18)	0.181	9.8 (12)	17.9 (10)	0.144

### Hypothesis Testing

We report on the findings for each hypothesis by number. Hypothesis 1: High fear of childbirth significantly increased the odds of having an epidural (OR = 2.02; 95% CI: 1.26-3.22; *p* = 0.003) controlling for maternal age, parity, infant macrosomia, previous cesarean section, fatigue, anxiety, sleep deprivation and available support (see Table 3). Adding fear of childbirth in the second step of the logistic regression model increased the Nagelkerke *R*^2^ from 0.17 to 0.19. In other words, adding fear of birth accounted for an addition 2% of the variance explained by the model.

**Table 3:** Hierarchical logistic regression model, testing predictors of epidural anesthesia

	**Beta**	**Wald**	**SE**	**p**	**OR**	**95 % CI**
**Step 1**						
Age > 35	−0.048	0.038	0.249	0.846	0.953	0.585-1.553
Multiparity	−1.482	36.896	0.244	< 0.001	0.227	0.141-0.366
Infant macrosomia	0.375	2.213	0.252	0.137	1.455	0.888-2.385
Previous caesarean section	−0.781	2.646	0.480	0.104	0.458	0.179-1.174
Fatigue	−0.017	2.271	0.011	0.132	0.984	0.963-1.005
Sleep deprivation	0.262	1.224	0.237	0.269	1.300	0.817-2.070
Support	−0.036	0.633	0.045	0.426	0.965	0.884-1.053
**Step 2**						
High fear	0.702	8.646	0.239	0.003	2.018	1.264-3.223
High anxiety	−0.089	0.124	0.254	0.725	0.915	0.556-1.504

Hypothesis 2: High anxiety was not a significant predictor of having an epidural (OR = 0.92; 95% CI: 0.56-1.50; *p* = 0.725) when controlling for parity, infant macrosomia, previous cesarean section, fatigue, fear of birth, sleep deprivation, and available support.

Hypothesis 3a: Fear of birth was not a significant predictor of caesarean section among nulliparas (OR = 1.58; 95% CI: 0.52-4.83; *p* = 0.421) when controlling for maternal age over 35, infant macrosomia, intent to request caesarean section, fatigue, anxiety, sleep deprivation, and available support.

Hypothesis 3b: When controlling for maternal age, infant macrosomia, previous caesarean section, history of difficult or upsetting labours and births, fatigue, anxiety, sleep deprivation, and available support fear of birth was not a significant predictor of caesarean section among multiparas (OR = 1.58; 95% CI: 0.52-4.83; *p* = 0.421).

Hypothesis 4: Sleep deprivation was not a significant predictor of caesarean section (OR = 1.16; 95% CI: 0.72-1.88; *p* = 0.540) when controlling for maternal age, infant macrosomia, intent to request caesarean section, previous caesarean sections, childbirth fear, anxiety, and available support.

Hypothesis 5: When controlling for maternal age, infant macrosomia, intent to request caesarean section, previous caesarean sections, childbirth fear, anxiety, sleep deprivation, and available support fatigue was not an independent significant predictor of caesarean section (OR = 0.98; 95% CI: 0.96-1.00; *p* = 0.154).

Hypothesis 6: Childbirth fear was not a significant predictor of assisted vaginal delivery (OR = 1.10; 0.56-2.17; *p* = 0.785) when controlling for maternal age, infant macrosomia, fatigue, anxiety, sleep deprivation, and available support.

Hypothesis 7: Anxiety was not a significant predictor of assisted vaginal delivery (OR = 1.16; 0.57-2.34; *p* = 0.686) when controlling for maternal age, infant macrosomia, fatigue, childbirth fear, sleep deprivation, and available support.

Hypothesis 8: When controlling for maternal age, fatigue, anxiety, sleep deprivation, and available support fear of birth was not a significant predictor of adverse neonatal outcomes (OR = 1.16; 95% CI: 0.52-2.58; *p* = 0.714).

Hypothesis 9: Anxiety was not a significant predictor of adverse neonatal outcomes (OR = 1.73; 95% CI: 0.76-3.92; *p* = 0.189) when controlling for maternal age, fatigue, fear of birth, sleep deprivation, and available support).

## Discussion

Our findings associating high fear of childbirth with multiparas viewing health as a stressor are supported by Laursen and colleagues who found a population-based cohort of nulliparous women with poor health were more likely to report fear of childbirth [44]. The prevalence of difficult and upsetting labours and births for multiparas with high childbirth fear reported in our study has been supported by other studies. In a sample of Chinese women who changed their preference to caesarean section for mode of birth, the largest proportion indicated fear of childbirth as their reason [45]. Of those in Pang’s group preferring caesarean section for their next birth, 14% reported a hemorrhage, 5% reported manual removal of placenta, and 9% reported vaginal instrumental birth during their previous births [45]. All of those events could contribute to women’s perceptions of upsetting and difficult labours and births.

Because significantly more multiparas in our high fear group reported previous difficult labour and birth experiences, it is not surprising that more of those women selected obstetricians as care providers compared to multiparas in the reference group. Our findings suggest those women anticipated more requirements for intervention in their births. Fisher and colleagues referred to retrospective horror where women described negative or difficult birth experiences inducing fear of upcoming births [46]. More British mothers experiencing a previous caesarean section or instrumental delivery reported fear of future birth than those with uncomplicated vaginal deliveries [47]. The literature that examines Scandinavian women’s childbirth fear and preference for caesarean section refutes our finding that high fear of birth was not significantly associated with a request for a caesarean section [18,29]. It is possible that the W-DEQ was less culturally sensitive to Canadian women’s experience of childbirth fear than it has been for Scandinavian women. We also selected the cut-off score for high fear (≥66), as suggested by Zar [37], but other researchers have used a cut-off score for severe childbirth fear (≥ 84) to predict risk for emergency caesarean section [21].

Our finding that high childbirth fear predicted use of epidural anaesthesia (EA) makes an important contribution to the literature. Many women who are fearful of birth are particularly afraid of the pain of labour [48-50]; however, there is not a clear link between fear of birth and pain relief during labour likely because pain relief is a complex matter. For 46 women surveyed 6 months after vaginal birth, those who chose epidural anaesthesia were more likely to report high fear of childbirth, an external locus of control for childbirth, and passive compliance in the birth process [51]. Some studies conducted in Scandinavia have reported fear during the first phase of labour predicts total amount of pain relief during labour and increased likelihood of receiving epidural analgesia [52,53]; however, a study of 47 nulliparous women found prenatal fear of childbirth was not associated with receiving an epidural [54]. A recent Swedish study reported women who were not successfully treated for childbirth fear were more likely to use epidurals for pain relief than women with no reported fear of childbirth [55].

Melender suggests caregivers ask women about their fears, provide opportunities to discuss them, and pay special attention to primiparas and multiparas reporting negative experiences of earlier pregnancies and births [56]. Psychosomatic support provided by caregivers can reduce women’s fear and concerns during pregnancy [22]. For example, nurses and physicians can spend time with women shortly after their birth experiences to listen to their birth stories and assist them to express any concerns or negative feelings. Having obstetrical caregivers provide psychosomatic support directly to women may strengthen women’s trust in the birth process and their care providers more effectively than referral to counselling; a recent study that compared Swedish women referred to a unit to treat childbirth fear (cognitive-behavioural therapy and psychoeducation) found the treated women experienced higher levels of obstetrical interventions (e.g., emergency caesarean section and induction of labour) than women in a reference group who did not report childbirth fear [57].

The evidence for effects of epidurals on mode of delivery is inconclusive. Epidurals have been associated with increased risk for caesarean section in some studies [58-60] but, in others, they have protected nulliparas from caesarean deliveries [61]. Sharma and colleagues found advancing maternal age and epidural anaesthesia increased the risks of unplanned abdominal delivery for nulliparas [60]. In a prospective cohort study, Nguyen and colleagues reported epidural use was associated with twice the risk for caesarean delivery for nulliparas and multiparas [59]. Fenwick et al. failed to find an association between high childbirth fear and epidural anaesthesia, after controlling for possible confounders in a multivariate model [58].

Despite reports that fear of birth often leads to emergency caesarean section, studies of the effect of childbirth fear on mode of delivery have been inconclusive. Serious fear of childbirth (a score of 84 or higher on the W-DEQ) at 32 weeks gestation in a sample of 1981 Swedish women tripled the risk of emergency caesarean section after excluding women with pregnancy complications and other possible confounders from the analysis [21]. On the other hand, British women’s (n = 433) fear of birth and anxiety were not associated with subsequent emergency caesarean sections [30] and Australian women’s childbirth fear was not significantly associated with emergency caesarean section when controlling for obstetrical complications [58].

Our study replicates findings that prenatal anxiety does not have a significant effect on mode of delivery and use of epidural anaesthetic in the first stage of labour [62]. The lack of association between prenatal anxiety and increased risk for epidural analgesia in our study is not in agreement with a study suggesting women with higher prenatal anxiety were more likely to receive analgesia and those who received analgesia more likely to have a surgical delivery [26] and a Swedish study that found worried women had significantly greater odds of an emergency caesarean section [33]. The lack of congruence might be explained by our use of state anxiety as a general measure of prenatal stress whereas Saunders and colleagues included pregnancy-specific anxiety, prenatal life events, state anxiety, and perceived stress and the Swedish study used references in an electronic database to worried and worrying [33].

Unlike other studies [15,17,33], our hypotheses about fatigue and sleep deprivation predicting increased risk for caesarean section were not supported. We controlled for many factors, including age, intent to request caesarean section, childbirth fear, and anxiety, whereas other investigators controlled only for infant birth weight [17] or age, multiple gestation, history of preterm births, and abdominal operations during pregnancy [15] or age, diabetes, gestational age, and epidural anaesthesia [33]. The women in those studies may have intended to request caesarean section, which was not investigated. According to Peterson, the reliability coefficients reported in our study correspond with the minimally acceptable reliability levels for applied research, as suggested by Nunnally, specifically 0.90 or greater [63]. Peterson suggested scales exhibiting very high alpha coefficients, e.g. 0.90 or higher, may imply a high level of item redundancy rather than scale reliability and scales with higher alphas are generally self-administered rather than interviewer-administered [63].

### Limitations

The study has a number of limitations. We did not use an objective measure of maternal sleep hours. Collecting objective actigraphic data about women’s sleep would have been very expensive and difficult to coordinate across large geographic distances. We sought no detail about women’s report of histories of difficult labour and births. We could have provided a short open-ended question to ask women to describe the nature of their experiences. When we divided the sample into multiparas and nulliparas to test our hypothesis about childbirth fear and caesarean birth we reduced the power to detect differences. The prevalence of adverse neonatal outcomes was very low; despite combining the outcomes in a composite measure the prevalence of any adverse outcome was 6.3%. The low prevalence of adverse neonatal outcomes reflected the health of the study participants. In retrospect, we could have removed the hypotheses about adverse outcomes arising from childbirth fear and anxiety from the suggested hypotheses because evidence was not as robust for negative neonatal outcomes arising from these psychological states. The study results are located in a Canadian context and are not generalizable to women in other countries with very different obstetrical care patterns and health care systems.

## Conclusion

Our results suggest attention be given to ameliorating women’s childbirth fear to reduce their exposure to epidural anaesthesia and to interventions for multiparous women to reduce childbirth fear arising from previous birth experiences. Effective interventions to reduce women’s exposure to caesarean section require particular attention to sources of women’s childbirth fear, particularly those women who have had experiences of difficult and upsetting labour and birth. Providing multiparous women with opportunities to describe their birth experiences and receive support around processing the events would be useful. Interventions to reduce trauma and fear arising from previous birth events could be evaluated through a randomized controlled trial. More prospective studies with large sample sizes from a variety of countries are required to explain how prenatal psychological states and sleep contribute to obstetrical interventions and potential adverse birth outcomes.

## Competing interests

The authors declare that they have no competing interests.

## Authors’ contributions

WH was the principal investigator for the grant, designed and conceived the study, obtained statistical support for the project, contributed to the analysis of the data, interpreted the data, and wrote the first draft of the paper. KS combined the datasets from the original study and PSBC, conducted preliminary statistical analyses, and contributed to interpretation of the data and drafts of the manuscript. EH and HB contributed to the design and conception of the study, interpretation of the data, and drafts of the manuscript. All authors read and approved the final manuscript.

## Pre-publication history

The pre-publication history for this paper can be accessed here:

http://www.biomedcentral.com/1471-2393/12/78/prepub
